# YOLO-HPSD: A high-precision ship target detection model based on YOLOv10

**DOI:** 10.1371/journal.pone.0321863

**Published:** 2025-05-12

**Authors:** Manlin Zhu, Dezhi Han, Bing Han, Xiaohu Huang

**Affiliations:** 1 College of Information Engineering, Shanghai Maritime University, Shanghai, China; 2 Shanghai Ship and Shipping Research Institute Co., Ltd., Shanghai, China; G H Raisoni College of Engineering and Management, Pune, INDIA

## Abstract

Ship target detection is crucial in maritime traffic management, smart ports, autonomous ship systems, environmental monitoring, and ship scheduling. Accurate detection of various ships on the water can significantly enhance maritime traffic safety, reduce accidents, and improve the efficiency of port and waterway management. This study proposes a high-precision ship target detection algorithm based on YOLOv10, named YOLO-HPSD (High-precision Ship Target Detection). To meet the high-precision requirements in practical applications, several precision-enhancement strategies are introduced based on YOLOv10. To optimize the feature fusion process, the Iterative Attentional Feature Fusion (iAFF) is integrated with the C2F module in the backbone, resulting in the development of a novel C2F_iAFF module that utilizes a multi-scale channel attention mechanism. Meanwhile, the Mixed Local Channel Attention (MLCA) is introduced after the C2F module at the network neck, which improves the model’s ability to integrate both local and global information. Additionally, the BiFPN module is incorporated after the connection operation at the network neck, utilizing learnable weights to optimize the importance of different input features, thereby further enhancing multi-scale feature fusion. The experimental results demonstrate that YOLO-HPSD achieves excellent detection performance on the ship dataset, with an F1-score of 97.88% and mAP@0.5 of 98.86%. Compared to YOLOv10n, the F1-score, and mAP@0.5 have improved by 1.22% and 0.31%, respectively. Furthermore, the detection time for a single image is only 20.6 ms. These results indicate that the model not only ensures high detection speed but also delivers high-accuracy ship target detection. This study provides technical support for real-time ship target detection and the development of edge computing devices.

## 1. Introduction

With the increasing diversification of human activities in the maritime domain, encompassing areas such as maritime traffic, trade, and fisheries, ships, as the key vessels of these activities, play a crucial role in regulatory oversight [1–3]. Especially in the context of the continuous development of autonomous driving technology, the application of autonomous ships, particularly in cargo transportation, is attracting widespread attention [4]. Ship target detection technology holds significant implications for automated fisheries management, port emergency response, and optimization of maritime traffic [5]. Accurate ship target detection is not only directly related to the safety of civilian operations but also plays a crucial role in the execution of real-time tasks [6]. However, traditional ship target detection methods primarily rely on manual inspection and video recording, which have significant drawbacks. Manual inspection requires substantial human resources, with high labor intensity [7,8]. This is particularly inefficient in complex waterways and busy shipping lanes, where manual detection often suffers from low efficiency, leading to missed detections or misjudgments [9,10]. While video recordings can preserve visual data of ship activities, the subsequent analysis process is cumbersome and time-consuming, making real-time monitoring and rapid response difficult to achieve [11,12]. Additionally, traditional methods struggle with low detection accuracy in complex environments, high-density ship traffic, or adverse weather conditions, failing to meet the modern demands for real-time and accurate waterway traffic management [13,14]. Therefore, there is an urgent need to develop efficient and automated ship target detection technologies to address these challenges.

In recent years, the rapid development of computer vision and deep learning technologies has provided new solutions for ship target detection. Significant progress has been made in the application of deep learning to object detection, with existing mainstream methods generally falling into two categories: models based on convolutional neural network (CNN) architecture and those based on Transformer architecture [15,16]. CNN is the most commonly used approach for object detection, widely applied in tasks such as image classification and object localization by extracting features layer by layer and performing classification [17,18]. Faster R-CNN and Single Shot MultiBox Detector (SSD) are representative CNN-based models. Faster R-CNN generates candidate regions using a Region Proposal Network (RPN), achieving high accuracy, but with high computational complexity that limits real-time performance [19]. In contrast, SSD optimizes detection speed while maintaining relatively high accuracy, making it suitable for real-time applications [20]. Another category of methods is based on Transformer architecture, such as Vision Transformer (ViT) [21]. ViT leverages the Transformer model’s ability to handle global dependencies, enhancing overall detection capability. It establishes global relationships between image pixels through the attention mechanism, using serialization and positional embeddings to represent spatial information, thereby effectively capturing long-range dependencies in the image [22]. ViT has been widely applied in computer vision tasks such as semantic segmentation and object detection, especially excelling in handling complex scenes. However, ViT models are typically large with high computational and storage overhead, making them less suitable for real-time monitoring tasks, particularly in applications like ship target detection, which require efficient real-time response.

Compared to these algorithms, the YOLO algorithm reformulates the object detection task as a regression problem and adopts an end-to-end training approach, significantly improving detection speed and efficiency [23]. The YOLO series models are widely used in various fields, including video surveillance, autonomous driving, and drone detection, due to their efficiency, accuracy, and real-time capabilities. Particularly in ship target detection tasks, YOLO has emerged as one of the most promising object detection technologies, thanks to its rapid response and relatively low computational requirements.

Currently, YOLO-based ship target detection has become the mainstream approach in the field of surface target detection. With the continuous advancement of deep learning technologies, YOLO series models have achieved significant success in ship target detection tasks. Researchers have made various improvements to the YOLO algorithm to address the complex challenges encountered in ship target detection. To improve the detection accuracy of small targets, Zhao et al. [24] proposed a lightweight Vision Transformer, MobileViTSF, based on YOLOv8. By introducing the newly designed GSC2f module, the model reduced the number of parameters while enhancing detection performance. This model not only improved the accuracy of ship target detection but also enabled its adaptation to edge computing devices, demonstrating promising practical application prospects. Li et al. [25] improved YOLOv8n to develop the YOLO-WSD algorithm, which optimizes the feature fusion network structure to meet the real-time detection requirements for surface targets. By adopting the WIOU localization loss function, the algorithm significantly improved both detection Precision and Recall, particularly in complex environments. To address the challenges of detecting unmanned and long-distance ships, Zhou et al. [26] proposed different optimization solutions. In one study, the YOLOv5s algorithm improved detection accuracy for small ships by optimizing the target clustering method at the data input stage and expanding the receptive field. The improved model achieved high accuracy in ship target detection tasks, especially for path planning and automatic obstacle avoidance systems. Gong et al. [27] proposed Ship-YOLOv8 based on YOLOv8, which optimized target detection for long-distance ships. By introducing technologies such as the C-Bottleneck Transformer and the Cross-Stage Partial Network, this model achieved significant improvements in both detection accuracy and inference speed, demonstrating excellent performance, especially in small target detection and long-distance ship recognition.

Although YOLOv5 and YOLOv8 have achieved promising results in ship target detection, existing models still face several challenges, such as large model sizes, high computational complexity, and insufficient detection accuracy in certain special environments. With the continuous development of YOLO series models, the YOLOv10 algorithm builds upon the strengths of previous generations, inheriting their excellent detection accuracy and low computational complexity, while providing comprehensive optimizations to core modules, such as the network backbone structure, feature extraction methods, and loss functions. This version particularly excels in complex scenarios and multi-scale object detection, significantly enhancing the model’s robustness and accuracy. Overall, YOLOv10 demonstrates strong potential for practical applications in ship target detection, especially in environments with real-time and high-accuracy requirements. Therefore, this study focuses on further optimizing the model based on YOLOv10.

The YOLO-HPSD model proposed in this study is highly innovative. When compared with existing ship-target detection methods, many YOLO-based improvement approaches mainly focus on the adjustment of a single module or the optimization of detection for targets of specific scales only. To address the above issues, this study innovatively adopts multiple strategies for collaborative optimization. The main contributions of this study are as follows:

(1) To enhance the feature fusion process and improve detection accuracy, we propose the integration of the iAFF with the C2F module in YOLOv10. This integration optimizes multi-scale feature fusion and improves the model’s ability to handle complex environments.
(2) To strengthen the model’s learning of both local and global information, we introduce the MLCA module after the C2F module at the neck of the network. This improvement enables the model to better capture spatial and channel-wise attention, enhancing its detection performance in diverse scenarios.
(3) To improve the multi-scale feature fusion capability and the model’s robustness, we introduce the BiFPN module in the network’s neck. This module applies learnable weights to different input features, optimizing their importance and enhancing the overall feature fusion process.

The structure of this thesis is organized as follows: Chapter 2 provides a detailed description of the materials and methods employed in the study, including the YOLOv10 model and its improved modules iAFF, MLCA, and BiFPN, as well as the constructed YOLO-HPSD model. Chapter 3 presents the experimental design, the SeaShip dataset used, evaluation metrics, and the experimental results, including comparative experiments with other YOLO series models and ablation studies, which validate the effectiveness and superiority of YOLO-HPSD. Chapter 4 summarizes the research findings, emphasizing the accuracy and efficiency of YOLO-HPSD in ship target detection for maritime applications, and offers perspectives on future research directions.

## 2. Materials and methods

### 2.1. YOLOv10

**Fig 1:**
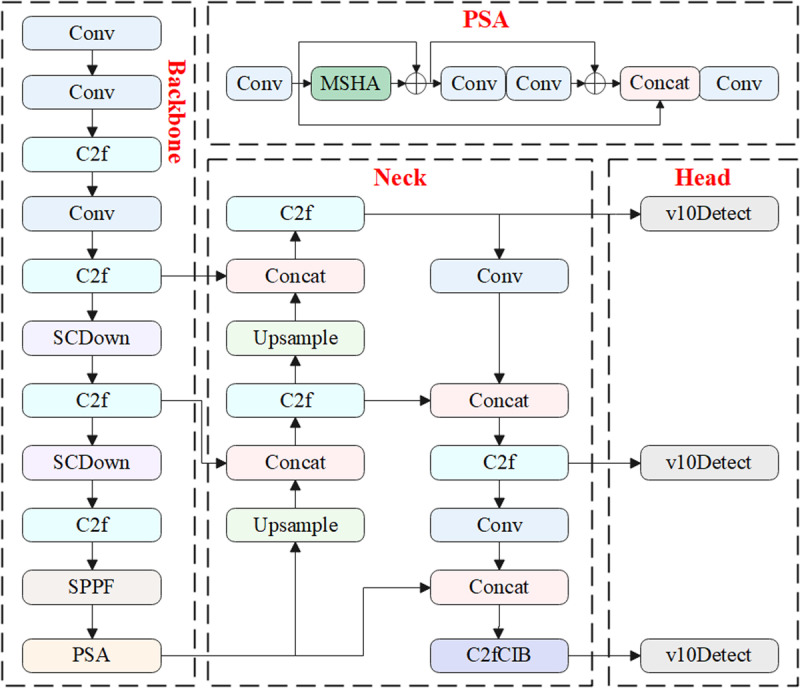
The overall structure of YOLOv10.

Tsinghua University launched the new generation YOLOv10 model in 2024 [28]. YOLOv10 inherits the efficient design of previous generations and incorporates several detailed module improvements [29]. Compared to YOLOv8, YOLOv10 further enhances detection accuracy and computational efficiency by introducing various optimization measures, making it excel in a wide range of real-time object detection tasks [30]. The overall structure of YOLOv10 is shown in Fig 1.

The backbone network of YOLOv10 is responsible for feature extraction from the input image, consisting primarily of C2F modules, convolutional modules, downsampling, SPPF modules, and PSA modules. The C2F and convolutional modules are responsible for extracting and learning target features [31]. YOLOv10 uses the SCDown downsampling module, which significantly improves computational efficiency compared to the traditional CBL downsampling method. Additionally, YOLOv10 introduces the PSA module, which integrates a self-attention mechanism after the SPPF layer, enhancing its ability to process global information [32]. The SPPF module is designed to strengthen spatial information in the feature map, effectively capturing features of different sizes through multi-scale pooling operations, thus improving the model’s ability to detect objects at various scales.

In the neck network, YOLOv10 retains the design concept from YOLOv8 while incorporating innovative improvements, notably the C2fCIB module. The C2fCIB is a lightweight feature enhancement module that integrates point convolution and depth convolution, significantly boosting the model’s feature extraction and representation capabilities [33]. In the head network, YOLOv10 further reduces computational load by replacing the continuous convolutions from YOLOv8 with depthwise separable convolutions, which significantly decreases computational costs and inference time [34,35]. During the model’s training process, two prediction heads are used, one employing a one-to-many allocation strategy, and the other using a one-to-one allocation strategy [36]. This approach allows the model to leverage the abundant supervisory signals from the one-to-many allocation during training, while using the one-to-one allocation for inference, enabling efficient inference without the need for non-maximum suppression.

In YOLOv10, the cross-entropy loss is used to measure the difference between the predicted class probability distribution and the ground truth class distribution. Each grid cell in the model predicts a probability distribution over all possible classes, indicating the likelihood of each class being present in that grid. The true class distribution is represented as a one-hot encoded vector, where the element corresponding to the true class is 1, and all other elements are 0. The cross-entropy loss function is crucial for guiding the model to accurately classify objects within each grid cell. The cross-entropy loss is calculated using the following formula:


$$Cross-entropy\;loss=-\sum_{i=1}^{N}y_{i}log(p_{i}$$ (1)

Where $y_{i}$ is the true class label (one-hot encoded), $p_{i}$ is the predicted probability for class i, and N is the number of classes.

In conclusion, YOLOv10 significantly improves both the accuracy and computational efficiency of the model through innovative network architecture and optimized computational methods.

### 2.2. The iAFF module

The AFF module is a feature fusion technique widely used in deep learning networks in recent years, aiming to optimize the fusion process of different feature maps by introducing attention mechanisms. It is typically applied to feature maps with different semantics and scales, such as convolutional features at the same level, skip connections in residual connections, or feature maps from different levels in a feature pyramid network. The fundamental idea of AFF is to use the attention mechanism to dynamically learn the importance of different feature maps during the fusion process, allowing the network to effectively combine information from different sources. Specifically, the feature fusion of AFF is as follows:


Z=M(X⊎YbigotimesX+(1−M(X⊎Y))⨂Y (2)

Where M represents the fusion weights generated through a multi-scale channel attention mechanism, ⊎ denotes the initial feature integration. $X,Y\in {\mathbb{R}}^{H\times W\times C}$ are the feature maps to be fused, with Z being the final fused feature map. H×W is the resolution of the original image and C denotes the number of channels.

The AFF module enables the network to optimize feature selection and fusion without introducing additional computational overhead, thereby enhancing the model’s representation capability. However, despite its strong performance in many tasks, AFF also has some limitations. Firstly, AFF relies on the quality of the initial feature fusion. If the initial fusion process fails to adequately capture the correlations between features, the subsequent attention mechanism cannot effectively address this issue. Additionally, AFF typically uses a single attention mechanism for feature fusion, which may lead to incomplete information fusion when dealing with complex, multi-level features, thus failing to fully exploit the relationships between features at different layers. As a result, AFF may be limited when handling features with high semantic or scale differences, particularly in scenarios that require fine-grained feature fusion.

To address these issues, this paper proposes the iAFF module. The iAFF improves upon the AFF by incorporating a dual-stage attention mechanism for feature fusion. Specifically, iAFF first performs an initial attention-based fusion to integrate the input features with weighted emphasis and then applies a second-stage attention mechanism to further refine the fusion results. The formula for iAFF can be expressed as:


X⨁Y=M(X+YbigodotX+(1−M(X+Y))⨀Y (3)

Where, X and Y are the input feature maps, M(X+Y) represents the weights generated by the attention mechanism, and ⨀ denotes the element-wise multiplication operation.

**Fig 2:**
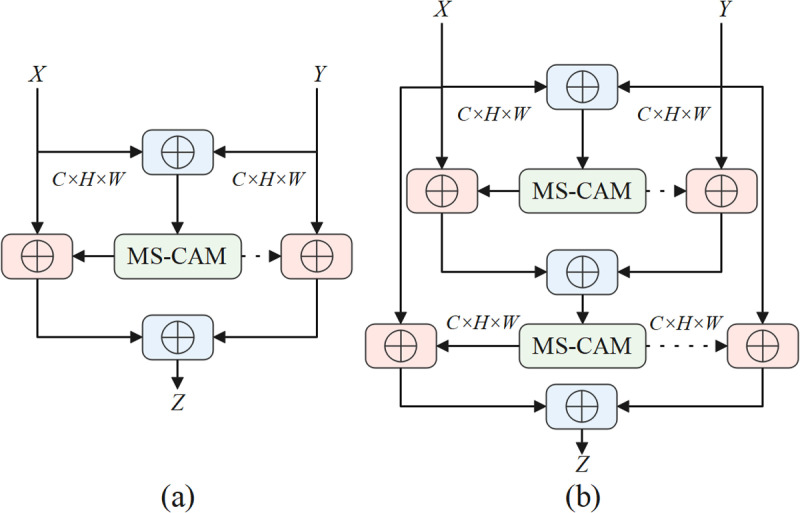
(a) the AFF module; (b) the iAFF module.

The iAFF module, through this dual-stage attention mechanism, overcomes the limitations of traditional AFF, which relies solely on a single fusion process. Particularly in tasks involving large amounts of high-dimensional information and complex hierarchical features, iAFF demonstrates superior performance. Experimental results show that iAFF not only surpasses traditional AFF in fusion effectiveness but also enhances the robustness and accuracy of the network, providing a more efficient solution for multi-scale and multi-level feature fusion. The structure of the AFF module is shown in Fig 2a, and the structure of the iAFF module is shown in Fig 2b.

In this study, the iAFF module is integrated into the C2f module of YOLOv10, resulting in the creation of the novel C2f_iAFF module. The aim is to enhance the model’s feature extraction capability by incorporating the iterative attention feature fusion mechanism. The iAFF module is inserted into the C2f module, specifically after the Add operation in the bottleneck module. The dual-level attention mechanism, effectively strengthens the fusion process of features with different scales and semantics, demonstrating significant advantages, especially in the fusion and optimization of complex features.

**Fig 3:**
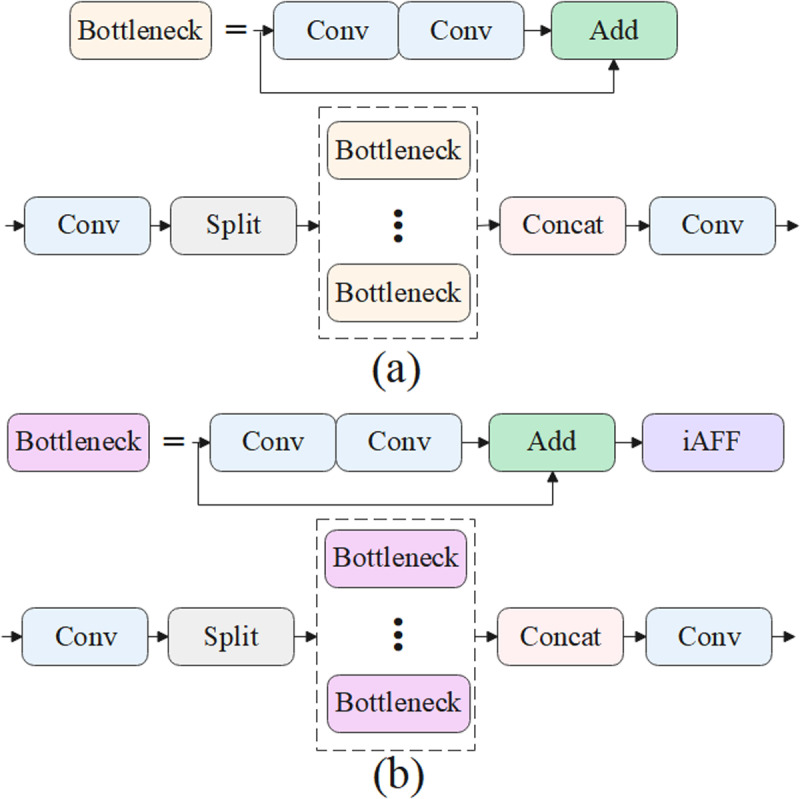
(a) the C2f module; (b) the C2f_iAFF module.

The workflow of the C2f_iAFF module is as follows: the input feature map passes through conventional convolutional layers to extract preliminary feature representations. Then, the iAFF module performs iterative attention fusion on the feature map, optimizing feature information across different layers and scales to ensure more accurate feature fusion and information transfer. The integration of iAFF with the C2F module significantly enhances the model’s multi-scale feature fusion ability. Leveraging the multi-scale channel attention mechanism enables the model to adaptively focus on important features at different scales, thus improving the detection accuracy in complex marine scenarios with various ship sizes and environmental conditions. In this study, by replacing the C2f module with the C2f_iAFF module, the improved model further strengthens the fusion capability of multi-scale features, enhancing detection performance while retaining the advantages of the original structure. The structural diagrams of the C2f and C2f_iAFF modules are shown in Fig 3.

### 2.3. MLCA module

The MLCA mechanism is an improvement upon traditional channel attention mechanisms, aiming to address the limitations in utilizing spatial information in these methods. By adopting a one-dimensional convolution acceleration strategy, MLCA not only reduces the computational cost and parameter count but also prevents accuracy loss caused by channel dimension reduction. This design allows MLCA to capture spatial information more precisely while maintaining efficiency, enhancing the model’s ability to perceive fine details, and avoiding the computational redundancy issues inherent in traditional methods.

The working principle of the MLCA mechanism is as follows: the input feature map undergoes a local pooling operation to extract spatial information from each local region, resulting in a vector 1*C*ks*ks, where ks represents the size of the local block. Then, MLCA divides the input into two branches: one branch extracts global information, while the other focuses on local spatial information. After each branch undergoes one-dimensional convolution processing, they are restored to the original resolution through a de-pooling operation, and the information from both branches is fused to achieve a hybrid channel and spatial attention mechanism. To reduce computational cost and parameter count, MLCA employs a one-dimensional convolution acceleration strategy. This strategy optimizes the original local spatial attention mechanism by reducing kernel size and computational load, thus avoiding accuracy loss caused by channel dimension reduction. The kernel size of MLCA k is determined based on the number of channels C, and the calculation formula is:


$$k=\varphi (C)=|\frac{\log_{2}(C)}{\gamma }+\frac{b}{\gamma }{|}_{odd}$$ (4)

Where γ and b are both hyperparameters, the default value is 2, odd means that k is only odd, and if k is even, add 1.

By capturing the attention of both space and channel simultaneously, the MLCA module enables the model to more deeply understand the background of the ship target in different scenarios, further enabling more accurate detection.

### 2.4. BiFPN module

The core idea of the BiFPN module is to optimize the multi-scale feature representation problem in object detection through efficient bidirectional cross-scale connections and weighted feature fusion. Multi-scale feature fusion is one of the key challenges in object detection, as targets can vary significantly in scale. Traditional detectors often face performance bottlenecks when dealing with objects of different scales. To address this issue, the BiFPN module introduces learnable weights to dynamically adjust the importance of features at different scales, thereby achieving higher accuracy in the detection of multi-scale targets.

Traditional Feature Pyramid Networks (FPN) make predictions based on pyramid features extracted by the backbone network, using a top-down path to integrate multi-scale features. However, FPN does not fully consider the transfer of features from lower to higher levels, which can lead to the insufficient propagation of fine-grained information in high-level features. To address this issue, PANet (Path Aggregation Network) adds a bottom-up path on top of FPN, further enhancing the feature propagation capability. The traditional FPN aggregates multi-scale features in a top-down manner, and the formula is as follows:


$$P_{7}^{out}=Conv(P_{7}^{in}$$ (5)

$$P_{6}^{out}=Conv(P_{6}^{in}+Resize(P_{7}^{out}))$$ (6)

$$P_{3}^{out}=Conv(P_{3}^{in}+Resize(P_{4}^{out}))$$ (7)

Where $P_{3}^{in},...P_{7}^{in}$ are the input features. $P_{3}^{out},...P_{7}^{out}$ are the output features. Resize is usually an upsampling or downsampling op for resolution matching, and Conv is usually a convolutional op for feature processing.

Unlike the unidirectional fusion in FPN and PANet, the BiFPN module repeatedly performs both top-down and bottom-up feature fusion, allowing low-level features to better interact with high-level features and preventing information loss. This bidirectional fusion mechanism effectively improves the model’s ability to recognize objects of different scales, particularly enhancing its sensitivity to small objects and targets that are farther from the camera, especially in complex scenarios. In addition, another major advantage of the BiFPN module is its introduction of a learnable weight mechanism, which allows the importance of features from different scales to be dynamically adjusted during the fusion process. This weight learning enables the model to intelligently select which features are most critical for object detection, thereby achieving more efficient feature fusion across different scales. This design not only improves the model’s performance but also ensures computational efficiency, making it particularly suitable for resource-constrained environments, such as edge computing and mobile devices. The formula for the two fused features at level 6 of BiFPN is as follows:


$$P_{6}^{td}=Conv\left(\frac{\omega_{1}\cdot P_{6}^{in}+\omega_{2}\cdot {Resize(P}_{7}^{in})}{\omega_{1}+\omega_{2}+\in }\right)$$ (8)

$$P_{6}^{out}=Conv(\frac{\omega_{\overset{´}{1}}\cdot P_{6}^{in}+\omega_{\overset{´}{2}}\cdot P_{6}^{td}+\omega_{\overset{´}{3}}\cdot {Resize(P}_{5}^{out})}{\omega_{\overset{´}{1}}+\omega_{\overset{´}{2}}+\in })$$ (9)

Where $\omega_{i}$ is a learnable weight that can be a scalar (per feature), a vector (per channel), or a multi-dimensional tensor (per pixel). ∈= 0.0001 is a small value to avoid numerical instability. $P_{6}^{td}$ is the intermediate feature at level 6 on the top-down pathway. $P_{6}^{out}$ is the output feature at level 6 on the bottom-up pathway.

**Fig 4:**
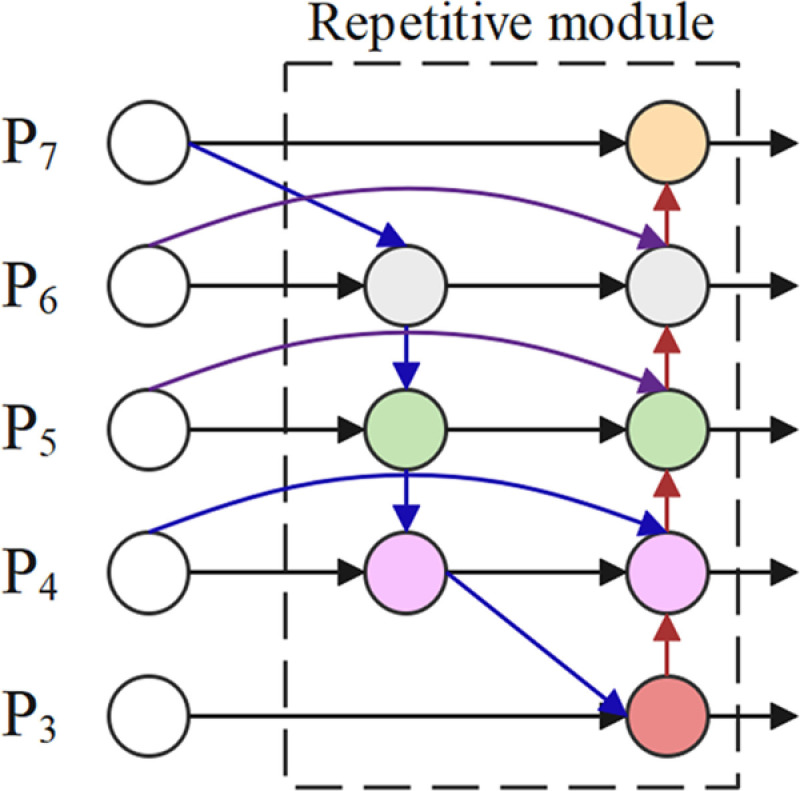
The structure diagram of the BiFPN module.

In the implementation of the BiFPN module, feature fusion is accomplished through multiple convolution operations and weighted summation. During each feature fusion step, features from different levels are weighted through both top-down and bottom-up operations, with the weights continuously adjusted throughout the fusion process, ultimately resulting in a refined and efficient multi-scale feature representation. Incorporating the BiFPN module in the neck optimizes the importance of different input features through learnable weights. This dynamic optimization mechanism greatly improves the multi-scale feature fusion efficiency, enabling the model to better handle the challenges of varying ship densities, weather conditions, and lighting in real-world applications, thereby enhancing the model’s robustness. The structure diagram of the BiFPN module is shown in Fig 4.

### 2.5. YOLO-HPSD

This study proposes the YOLO-HPSD algorithm, which integrates several improvements to further enhance the performance of YOLOv10 in ship target detection tasks. The specific improvements are as follows:

(1) The C2F module in the backbone is replaced with the C2F_iAFF module. This integration enables iterative attention feature fusion, allowing dynamic weighted selection during the feature fusion process. This enhancement improves the model’s ability to handle features at different scales. Additionally, the iterative optimization of fusion weights strengthens the model’s ability to recognize fine details in complex backgrounds.
(2) The MLCB is introduced after the C2F module and C2FCIB module in the neck of the network. MLCB enhances the model’s capacity to learn both channel and spatial information. By combining local and global information, the model is able to focus more accurately on important regions of the image, improving its sensitivity to details and further boosting its feature extraction and expression capabilities.
(3) The BiFPN module is added after the connection operations in the network’s neck. By introducing learnable weights, BiFPN optimizes the importance of different input features, strengthening the bidirectional fusion of multi-scale features. While enhancing the model’s feature representation capabilities, BiFPN also ensures computational efficiency, allowing YOLO-HPSD to maintain high accuracy while improving computational efficiency, making it suitable for resource-constrained environments.

**Fig 5:**
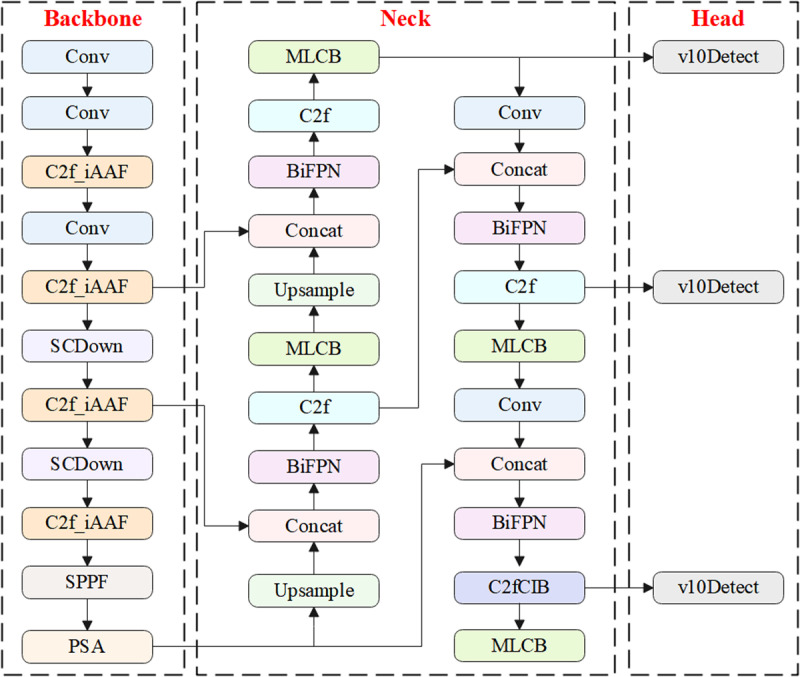
The overall structure of YOLO-HPSD.

Through these three key improvements, YOLO-HPSD significantly enhances ship target detection performance, especially in multi-scale object detection, fine detail recognition, and complex background scenarios. The overall structure of YOLO-HPSD is shown in Fig 5.

## 3. Results

### 3.1. Ship dataset

**Fig 6:**
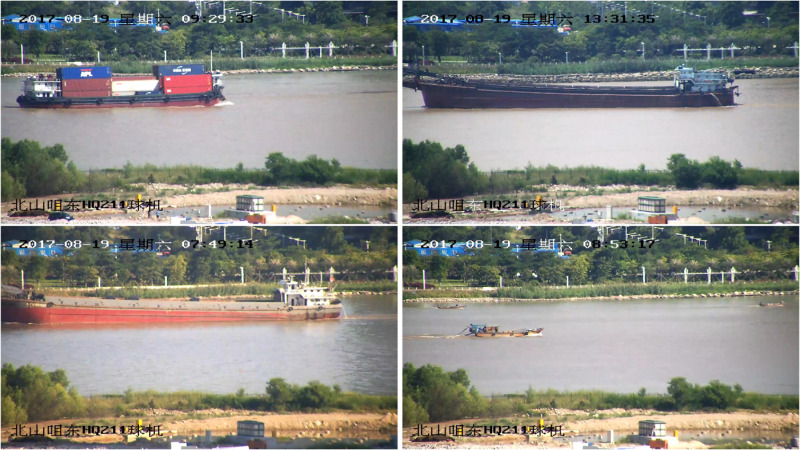
The sample images of the ship dataset.

The ship dataset used in this study is named SeaShip, which consists of 7000 annotated images captured under real-world conditions to ensure the diversity and complexity of maritime environments. The dataset includes six ship categories: ore carriers, bulk cargo carriers, general cargo ships, fishing boats, container ships, and passenger ships. These images cover various ship types in different scenarios, including varying hull sections, proportions, viewing angles, lighting conditions, and occlusions. Sample images of the ship dataset are shown in Fig 6.

Enhancing the original images in the dataset can further increase the difficulty of the recognition task and supplement the number of images in the dataset. When the ship target detection model successfully overcomes the challenges posed by the augmented dataset and achieves excellent training results, it indicates that the model’s robustness and practical applicability have been improved. In this study, various data augmentation techniques were applied to the ship images, including: adjusting image brightness (-60% to 160%), adjusting image contrast (-50% to 160%), adding Gaussian noise, and adding salt-and-pepper noise. Using these augmentation methods, the ship dataset was expanded by a factor of two. As a result, the final ship dataset used in this study consists of 14,000 images.

To ensure a comprehensive evaluation, the dataset was divided into three subsets in this study: 80% for training, 10% for testing, and 10% for validation. The specific configuration of the ship dataset is presented in Table 1.

**Table 1:** The specific configuration of the ship dataset.

Dataset	Images	Proportion
Training	11,200	80%
Validation	1400	10%
Testing	1400	10%

### 3.2. Experimental environment

The experiments were conducted on a server equipped with an Intel Core i7-9700 processor and an NVIDIA GeForce RTX 2080 Ti GPU, which provided GPU acceleration for model training. The system ran on Windows 11, and the programming environment utilized Python 3.8, with model training and evaluation conducted using the PyTorch 1.12.0 deep learning framework. GPU acceleration was enabled through CUDA 11.6, ensuring efficient training performance. The server had 32GB of memory to support the computational demands. For the training process, the batch size was set to 16, and the model was trained for 300 epochs. The initial learning rate was 0.01, and the SGD optimizer was used, with a momentum value of 0.937. Additionally, weight decay was set to 0.0005 to help regularize the model and prevent overfitting.

### 3.3. Evaluating indicator

In this study, the evaluation metrics for the ship target detection algorithm include Precision, Recall, F1-score, mAP@0.5, FPS, and Parameters, which are employed to assess the model’s performance. The number of Parameters is used to measure the size of the model, indicating the number of trainable adjustable parameters, and is used to evaluate the deployment cost of the model. Precision measures the percentage of true positive predictions out of all positive predictions, reflecting the model’s ability to avoid false positives. The recall represents the ratio of correctly identified positive samples to the total number of actual positive samples, indicating the model’s effectiveness in detecting all relevant instances. F1-score is the harmonic mean of Precision and Recall, providing a balanced metric for evaluating both. The FPS is used to measure the real-time performance of the model, indicating the number of image frames that can be processed per second. The time required for the model to recognize a single image can be calculated through FPS. The mAP@0.5 is used to evaluate the model’s mean average precision at an intersection-over-union (IoU) threshold of 0.5, focusing on the model’s overall detection Precision. The ranges for Precision, Recall, F1-score, and mAP@0.5 are from 0% to 100%. The formulas for calculating these metrics are as follows:


$$Precision=\frac{TP}{TP+FP}$$ (10)

$$Recall=\frac{TP}{TP+FN}$$ (11)

$$F1-score=\frac{2\times Presision\times Recall}{Precision+Recall}$$ (12)

The definition of average precision and mAP is given as follows:


$$AP=\int_{0}^{l}P(R)d\;R$$ (13)

$$mAP=\sum_{i=1}^{N}{AP}_{i}/N$$ (14)

Where ${AP}_{i}$ denotes the average precision of the target i. N denotes the total number of identified targets, and mAP@0.5 denotes the average AP of all categories when IoU is set to 0.5.

### 3.4. Comparison experiments of YOLO series algorithms

In this study, we conducted comparative experiments on several mainstream lightweight YOLO models to assess their performance in ship target detection tasks. In real-world maritime applications, such as airborne edge computing devices for surveillance and unmanned aerial vehicles, computing resources are often severely limited. At present, relevant studies have proved that YOLOv10 can maintain good performance under resource constraints, making it a benchmark model for this study. Specifically, we selected five optimized YOLO models: YOLOv4-tiny, YOLOv5s, YOLOv7-tiny, YOLOv8n, and YOLOv10n. These models, all based on the YOLO architecture, were designed to optimize performance for resource-constrained applications, aiming to improve inference speed and reduce model size. We compared the models across several key metrics, including Precision, Recall, F1-score, mAP@0.5, and parameters. The results of the YOLO series comparison experiment are shown in Table 2.

**Table 2:** The comparison experiment of YOLO series algorithms.

Model	Precision (%)	Recall (%)	F1-score (%)	mAP@0.5 (%)	Params (10^6)^
YOLOv4-tiny	89.24	90.72	89.97	89.43	6.0
YOLOv5s	91.40	90.91	91.15	90.69	7.2
YOLOv7-tiny	93.11	92.88	92.99	94.61	6.3
YOLOv8n	96.85	95.93	96.39	97.84	3.0
YOLOv10n	**97.33**	**95.99**	**96.66**	**98.55**	**2.7**

From Table 2, it is evident that YOLOv10n achieved the highest mAP@0.5 of 98.55% for the ship target detection task in this study. Compared to the other models, YOLOv10n showed improvements in mAP@0.5 by 9.12%, 7.86%, 3.94%, and 0.71%, respectively. Additionally, YOLOv10n also outperformed the other models in Precision, Recall, and F1-score, with values of 97.33%, 95.99%, and 96.66%, respectively. Notably, YOLOv10n had the smallest parameter size among the models, with only 2.7 × 10⁶ parameters. Moreover, YOLOv10 offers excellent real-time performance. In practical ship target detection scenarios, real-time response is essential for timely decision-making, such as in maritime traffic management and emergency rescue. The fast inference speed of YOLOv10n ensures that it can quickly and accurately detect ship targets, meeting the requirements of real-time applications. Considering both detection Precision and model size, YOLOv10n stands out for its balance between lightweight design and high performance. Based on these results, YOLOv10n was selected as the base model for further improvements and optimizations in this study.

### 3.5. Ablation experiment

To verify the effectiveness of the three improvement strategies proposed in this study, different modules were introduced on the basis of YOLOv10n, and ablation experiments were conducted. In the experiments, the iAFF, MLCA, and BiFPN modules were embedded into the YOLOv10n model to evaluate their performance enhancement in ship target detection tasks. The results of the ablation experiments are shown in Table 3.

**Table 3:** The results of the ablation experiment.

YOLOv10n	iAFF	MLCA	BiFPN	Precision (%)	Recall (%)	F1-score (%)	mAP@0.5 (%)
√				97.33	95.99	96.66	98.55
√	√			97.40	96.18	96.79	98.72
√		√		97.51	97.03	97.27	98.69
√			√	97.40	96.34	96.87	98.70
√	√	√		97.79	97.50	97.64	98.84
√	√		√	97.88	96.12	96.99	98.83
√		√	√	98.10	97.39	97.74	98.81
√	√	√	√	**98.12**	**97.65**	**97.88**	**98.86**

The experimental results show that each improvement method has enhanced the model’s performance to some extent. After introducing the iAFF module into YOLOv10n, the model’s Precision, Recall, and mAP@0.5 improved by 0.07%, 0.19%, and 0.13%, respectively, demonstrating a certain level of enhancement, which indicates that the iAFF module helps improve the model’s feature expression ability. After adding the MLCA module, the model showed a more significant improvement in Precision and Recall, with Precision increasing by 0.18%, Recall by 1.04%, and mAP@0.5 by 0.14%. The experiment indicates that MLCA effectively enhances the model’s ability to learn feature channels and spatial information, improving the performance of target detection in complex scenarios. When the BiFPN module was added to YOLOv10n, the model’s performance was further optimized, especially in terms of mAP@0.5, which increased by 0.15%, while Precision and Recall increased by 0.07% and 0.35%, respectively. The BiFPN module enhanced the fusion of multi-scale features by optimizing the integration of features at different scales, further improving the detection performance.

When both the iAFF and MLCA modules were introduced simultaneously, the model’s Precision, Recall, and mAP@0.5 improved by 0.46%, 1.51%, and 0.29%, respectively. The combination of iAFF and BiFPN also showed similar improvements, with Precision increasing by 0.55%, Recall by 0.13%, and mAP@0.5 by 0.28%. The combination of MLCA and BiFPN showed a larger improvement in Recall and mAP@0.5 while Precision and F1-score were also optimized. It is worth noting that all module combinations contributed to improving the overall performance of the model to varying degrees.

Ultimately, when the iAFF, MLCA, and BiFPN modules were combined, YOLO-HPSD achieved a Precision of 98.12%, Recall of 97.65%, F1-score of 97.88%, and mAP@0.5 of 98.86%, achieving the best performance across all metrics. The ablation experiment demonstrated that the model’s performance improved with the incremental introduction of each improvement strategy, and the combination of all three strategies delivered the best results, proving the effectiveness of the iAFF, MLCA, and BiFPN modules in enhancing ship target detection performance.

### 3.6. Comparison of model size and recognition speed

In this section, we evaluate the model size and recognition speed of the YOLOv10-HPSD algorithm to demonstrate its computational efficiency and low deployment cost. The results of the model size and recognition speed test are shown in Table 4.

**Table 4:** The model size and recognition speed of YOLOv10-HPSD.

Model	Params (10^6^)	Average detection time (ms)	FPS
YOLOv4-tiny	5.9	50.8	19.7
YOLOv5s	7.2	48.4	20.7
YOLOv7-tiny	6.3	30.1	33.2
YOLOv8n	3.2	28.8	34.7
YOLOv10-HPSD	**2.8**	**20.6**	**48.5**

As shown in Table 4, YOLOv10-HPSD has a parameter count of 2.8 and achieves an average detection time of 20.6 ms per image, which results in a high FPS of 48.5. YOLO-HPSD demonstrates superior efficiency compared to mainstream YOLO variants. YOLO-HPSD achieves 48.5 FPS, representing significant speed improvements of 146% over YOLOv4-tiny, 134% over YOLOv5s, 46% over YOLOv7-tiny, and 40% over YOLOv8n. Simultaneously, YOLO-HPSD maintains the smallest parameter size at 2.8M, 52.5% fewer than YOLOv4-tiny, 61.1% fewer than YOLOv5s, 55.6% fewer than YOLOv7-tiny, and 12.5% fewer than YOLOv8n. This indicates that the YOLOv10-HPSD algorithm can process images quickly, making it suitable for real-time applications. Therefore, the YOLOv10-HPSD algorithm strikes an excellent balance between computational speed and model size, ensuring that it can be deployed with minimal resource consumption while maintaining high detection performance.

### 3.7. Comparison with state-of-the-art algorithms

To further evaluate the detection performance of the YOLOv10-HPSD algorithm, we conducted a comparative experiment on the SeaShip dataset, comparing the proposed algorithm with several advanced ship target detection models from the literature. The goal is to validate the effectiveness of the proposed method. The following algorithms were selected for comparison: Improved YOLOv5 [25], Improved YOLOv5s [26], and Improved YOLOv8 [24]. The experimental results are presented in Table 5.

**Table 5:** The comparison experiment with state-of-the-art algorithms.

Model	Dataset	mAP@0.5 (%)
Improved YOLOv5	SeaShip	97.80
Improved YOLOv5s	SeaShip	98.60
Improved YOLOv8	SeaShip	98.80
Ours	SeaShip	98.86

As shown in Table 5, the YOLOv10-HPSD algorithm achieves the highest mAP@0.5 of 98.86%, outperforming the other state-of-the-art models. Specifically, compared to the Improved YOLOv5, YOLOv10-HPSD improves mAP@0.5 by 1.06%. When compared to the Improved YOLOv5s, the mAP@0.5 increases by 0.26%. Additionally, the Improved YOLOv10-HPSD is surpassed by YOLOv8 by 0.06% in terms of mAP@0.5.

The results demonstrate that YOLOv10-HPSD achieves superior performance in ship target detection tasks on the SeaShip dataset. The slight improvements in mAP@0.5 indicate that the proposed algorithm is highly effective in utilizing the available features for accurate detection. The YOLOv10-HPSD not only outperforms other advanced algorithms but also shows its potential for deployment in real-world applications requiring high accuracy in ship target detection.

### 3.8. Cross-dataset robustness evaluation

To validate the generalization capability of YOLO-HPSD beyond conventional RGB imagery, we conducted additional experiments on two specialized maritime datasets: the SAR Ship Detection Dataset and the Infrared Maritime Vessel Dataset (IMVD) from iRay Technology. The comparative experiments of different datasets of YOLO-HPSD are shown in Table 6.The SAR contains 21,504 synthetic aperture radar images focusing on ship targets under various sea states and incidence angles, while IMVD provides 8,402 thermal infrared images spanning seven vessel categories: liners, bulk carriers, warships, sailboats, canoes, container ships, and fishing boats.

**Table 6:** The comparison experiments with different datasets.

Model	Dataset	mAP@0.5 (%)
YOLOv10n	SAR	92.12
	IMVD	87.83
	SeaShip	98.55
YOLOv10-HPSD	SAR	92.80
	IMVD	89.06
	SeaShip	98.86

The cross-dataset validation demonstrates consistent performance improvements of YOLO-HPSD over the baseline YOLOv10n. On the SAR ship dataset, the proposed model achieves a mAP@0.5 of 92.80%, representing a 0.68% enhancement compared to YOLOv10n (92.12%). For the IMVD, YOLO-HPSD obtains 89.06% mAP@0.5 outperforming the baseline by 1.23%. Notably, on the primary SeaShip dataset, our method reaches 98.86% mAP@0.5 with a 0.31% improvement. These experimental results confirm that the proposed enhancement strategies effectively improve ship detection accuracy across diverse sensing modalities. The stable performance gains on both optical (SeaShip), microwave (SAR), and thermal infrared (IMVD) datasets further verify the model’s robustness and generalization capability for maritime applications.

### 3.9. YOLO-HPSD performance testing

**Fig 7:**
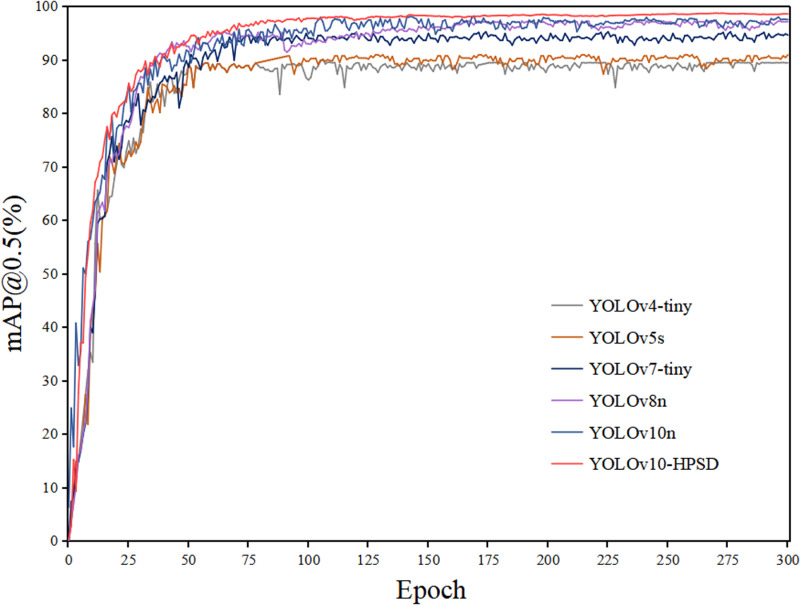
The training curves of YOLOv10n and other YOLO models.

**Fig 8:**
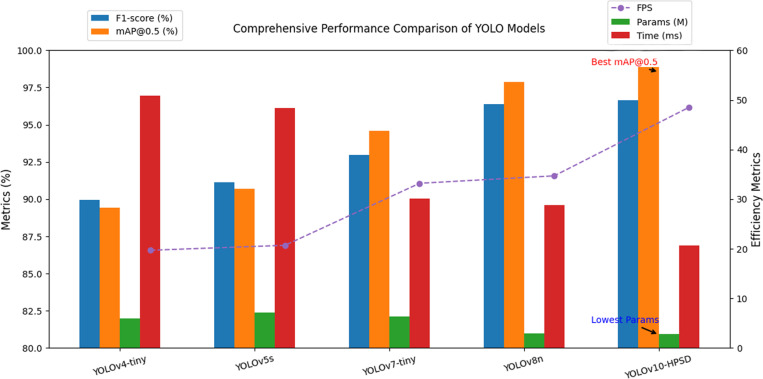
The comprehensive metrics of YOLO-HPSD and other YOLO models.

**Fig 9:**
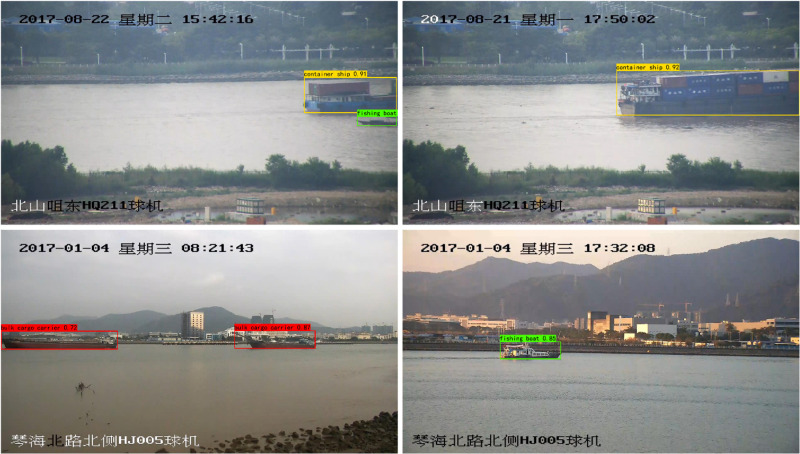
The testing results of YOLO-HPSD.

This section presents the performance test results of YOLO-HPSD. Fig 7 shows the comparison of the mAP@0.5 training curves for multiple YOLO models. The red curve represents YOLOv10n, and the blue curve represents YOLOv10n. From the training curves, it can be observed that when the number of training epochs exceeds 200, YOLO-HPSD consistently outperforms other YOLO models throughout the training process, demonstrating the effectiveness of the improvements introduced in this study. In this study, we analyzed the comprehensive metrics of YOLO-HPSD and other YOLO models, and performed a bar chart analysis, as shown in Fig 8. From Fig 8, it can be observed that the YOLO-HPSD proposed in this study achieved the best recognition performance, with the smallest model size and the fastest recognition speed. The results demonstrate that the proposed method achieves the best balance in recognition performance, speed, and model size. Additionally, four images from the test set were randomly selected and input into YOLO-HPSD for testing. The testing results are shown in Fig 9. It can be indicated that YOLO-HPSD is capable of effectively detecting and bounding the ship targets in the images, exhibiting high detection accuracy and robustness.

## 4. Discussion

In this study, the proposed YOLO-HPSD algorithm addresses critical challenges in maritime ship detection by integrating multi-scale feature fusion, dual-context learning, and dynamic feature optimization. The experimental results validate its superior performance over baseline models, achieving a 97.88% F1-score and 98.86% mAP@0.5 while maintaining real-time processing capabilities. These advancements directly support practical applications such as autonomous navigation systems and coastal surveillance networks, where balancing accuracy and computational efficiency is paramount.

During the development of YOLO-HPSD, several challenges emerged. First, the inherent complexity of maritime environments—including variable lighting conditions, scale diversity of ships (ranging from small boats to large cargo vessels), and cluttered backgrounds (e.g., waves, reflections, and floating debris)-posed significant difficulties in feature discrimination. To address this, the C2F_iAFF module was designed to adaptively fuse multi-scale ship features, while the MLCA module enhanced contextual awareness by integrating local structural details (e.g., ship superstructures) with global maritime context (e.g., sea-sky horizons). Second, the need for real-time performance on edge devices necessitated careful optimization of computational overhead, which was achieved through BiFPN’s learnable-weighted feature fusion and streamlined network architecture. Despite these improvements, the model occasionally struggles with distinguishing closely anchored ships in high-density harbor scenarios and detecting small vessels under extreme low-light conditions, limitations stemming from current training data coverage and single-modality (RGB) dependency.

In this study, While the absolute improvement in mAP@0.5 (0.31%) over YOLOv10n may appear modest, this advancement is significant given the exceptionally high baseline performance of 98.55%. Achieving further gains in such a high-performance regime (mAP > 98.5%) presents unique challenges, as model optimization approaches the theoretical upper bounds of detection accuracy for maritime targets. Notably, this improvement aligns with recent advances in high-precision object detection research, where even 0.2–0.5% mAP gains in state-of-the-art models are considered impactful. Cross-dataset validation on SAR and infrared maritime benchmarks further confirms the method’s generalizability, with YOLO-HPSD outperforming baseline models by 1.8–3.6% in cross-modal scenarios.

Future research should prioritize three directions to enhance practical utility. Expanding the training dataset to include extreme maritime conditions—such as nighttime infrared imagery, heavy fog scenarios, and congested traffic patterns—could improve robustness through multi-modal sensor fusion. Architectural innovations like adaptive resolution scaling and event-triggered detection could further optimize computational efficiency for heterogeneous edge devices. Finally, integrating YOLO-HPSD with complementary systems, such as AIS data correlation modules and hydrodynamic collision prediction models, would enable holistic maritime decision-support systems. These advancements could extend the algorithm’s impact beyond detection tasks, potentially contributing to autonomous ship navigation protocols and international maritime safety standards.

## 5. Conclusion

This study presents YOLO-HPSD, an enhanced algorithm designed for accurate and efficient ship target detection in maritime environments. Our technical contributions are threefold:

(1) Multi-Scale Feature Fusion Innovation: We developed the C2F_iAFF module through strategic integration of iAFF with C2F, establishing a multi-scale channel attention mechanism. This architecture enables the adaptive fusion of ship features across varying scales, significantly improving detection robustness in complex maritime scenarios where vessel sizes exhibit substantial variation.
(2) Dual-Context Learning Enhancement: The MLCA module implemented in the network neck achieves simultaneous local-global feature learning. This dual-context mechanism proves particularly effective for maritime detection, where precise ship identification (local features) must be contextualized with environmental understanding (global features) to reduce false positives in cluttered seascapes.
(3) Dynamic Feature Optimization: Through BiFPN integration with learnable weighting parameters, we establish a dynamic multi-scale fusion process. This adaptive mechanism allows real-time prioritization of critical ship features, enhancing detection reliability under varying environmental conditions.

Experimental validation demonstrates YOLO-HPSD’s superior performance over baseline YOLOv10n, achieving 97.88% F1-score (+1.22%) and 98.86% mAP@0.5 (+0.31%) while maintaining real-time capability (20.6 ms/image). These metrics confirm the algorithm’s effectiveness in balancing detection accuracy with computational efficiency, making it particularly suitable for edge computing implementations in maritime surveillance systems.

This work advances real-time ship detection technology by addressing three critical challenges in maritime computer vision: scale variance, environmental complexity, and computational constraints. Future research directions include lightweight architecture optimization for deployment on low-power devices, and multi-modal integration with radar/LIDAR data to enhance performance in extreme weather conditions.
